# Ketogenic Diet Benefits to Weight Loss, Glycemic Control, and Lipid Profiles in Overweight Patients with Type 2 Diabetes Mellitus: A Meta-Analysis of Randomized Controlled Trails

**DOI:** 10.3390/ijerph191610429

**Published:** 2022-08-22

**Authors:** Chong Zhou, Meng Wang, Jiling Liang, Guomin He, Ning Chen

**Affiliations:** 1 School of Journalism and Communication, Wuhan Sports University, Wuhan 430079, China; 2 Reproductive Medicine Center, Tongji Hospital, Tongji Medical College, Huazhong University of Science and Technology, Wuhan 430030, China; 3 Tianjiu Research and Development Center for Exercise Nutrition and Foods, Hubei Key Laboratory of Exercise Training and Monitoring, College of Health Science, Wuhan Sports University, Wuhan 430079, China; 4 School of Economics and Management, Wuhan Sports University, Wuhan 430079, China

**Keywords:** glycemic management, body weight control, very low-carbohydrate diet, glycated hemoglobin, high-density lipoprotein, therapeutic intervention

## Abstract

A ketogenic diet, characterized by low calories with high levels of fat, adequate levels of protein, and low levels of carbohydrates, has beneficial effects on body weight control in overweight patients. In the present study, a meta-analysis was conducted to investigate the role of a ketogenic diet in body weight control and glycemic management in overweight patients with type 2 diabetes mellitus (T2DM). In summary, we systematically reviewed articles from the Embase, PubMed, Web of Science and Cochrane Library databases and obtained eight randomized controlled trials for meta-analysis. The results show that a ketogenic diet had significantly beneficial effects on the loss of body weight (SMD, −5.63, *p* = 0.008), the reduction of waist circumference (SMD, −2.32, *p* = 0.04), lowering glycated hemoglobin (SMD, −0.38, *p* = 0.0008) and triglycerides (SMD, −0.36, *p* = 0.0001), and increasing high-density lipoproteins (SMD, 0.28, *p* = 0.003). Overall, these results suggest that a ketogenic diet may be an effective dietary intervention for body weight and glycemic control, as well as improved lipid profiles in overweight patients with T2DM. Hence, a ketogenic diet can be recommended for the therapeutic intervention of overweight patients with T2DM.

## 1. Introduction

Type 2 diabetes mellitus (T2DM) is a high-incidence chronic metabolic disorder, with high mortality and morbidity rates worldwide due to its multiple complications [1]. Patients with T2DM are more likely to suffer from cardiovascular diseases, diabetic neuropathy, and many other complications, as the major causes of diabetes-related deaths [2]. Although medication therapy, such as metformin, gliquidone, and acarbose, can reduce the fluctuation in blood glucose levels, lifestyle interventions, especially medical nutrition, are an effective, recommended, non-pharmacological intervention [3].

A ketogenic diet, characterized by low calories with high-level fat, moderate-level protein, and very low-level carbohydrate (usually less than 50 g/d), has been introduced as a nutrition-based intervention for the treatment of epilepsy through energy supply from ketone bodies, which mimics metabolic starvation due to the strict restriction of carbohydrates [4,5]. There are four types of ketogenic diet, including standard ketogenic diet, cyclical ketogenic diet, targeted ketogenic diet, and high-protein ketogenic diet [6]. Among them, standard ketogenic diet, which typically contains 70 percent fat, 20 percent protein and only 10 percent carbohydrates, is the most studied and recommended. Under the condition of reduced carbohydrate consumption, ketone bodies produced by the breakdown of fats in the liver, is an alternative source of energy to glucose, especially the central nervous system [7]. Recently, this diet has been reported to have great potential in the body weight control of patients with obesity [8,9,10]. A previous review has documented that obesity is a risk factor for T2DM and its complications, such as chronic cardiovascular diseases [11]. Although previous meta-analyses and systematic reviews have demonstrated the efficacy of a ketogenic diet in body weight control [12,13], conflicting findings have been reported with regard to changes in glycemic and lipid profiles of T2DM patients under this dietary intervention. A randomized controlled trial (RCT) reported that a ketogenic diet could mitigate insulin resistance and reduce glycemic responses, thereby improving the glycemic profiles of T2DM patients [14]. Conversely, other evidence indicates that a low-carbohydrate and high-fat diet is associated with deteriorated lipid profiles [15,16]. The mechanisms that underlie the associations between ketogenic diet and T2DM are still a subject of debate, and gut microbiota might play a significant role in the relationship between very low-carbohydrate ketogenic diet (VLCKD) and reducing obesity [17]. The appropriateness of ketogenic diets in overweight T2DM patients on body weight control, and glycemic and lipid profile management, is still not firmly established. Therefore, in the present study, to validate the effect of a ketogenic diet on overweight patients with T2DM, we conducted a meta-analysis based on comprehensive metabolic parameters including body weight changes, glycemic control, and lipid profiles of overweight patients with T2DM in the presence of ketogenic diet intervention, relative to other types of dietary interventions.

## 2. Materials and Methods

### 2.1. Data Sources and Search Strategy

This meta-analysis was conducted based on the standard Cochrane protocols. Briefly, two independent reviewers systematically searched for relevant literature in the PubMed, Embase, Cochrane Library, and Web of Science databases, according to the guidelines of the Preferred Reporting Items for Systematic Reviews and Meta-analyses (PRISMA). Specifically, we targeted RCTs that investigated and evaluated the effects of a ketogenic diet on body weight change, glycemic control, and lipid profile in overweight T2DM patients, which were published until 30 April 2022. To identify relevant studies, the reviewers used the following search terms: (“Diet, Ketogenic” [MeSH] OR “Ketogenic*” [Title/Abstract]) AND (“Diabetes Mellitus, Type 2” [MeSH] OR “Non-Insulin-Dependent Diabetes*” [Title/Abstract] OR “Type II Diabetes*” [Title/Abstract]) AND (“Overweight” [MeSH] OR “Obesity” [MeSH]). The search filter was set as “clinical trials”, and eligible RCTs were also obtained from reference lists of relevant review articles. A ketogenic diet was defined as a dietary intake comprising high fats, moderate proteins, and very low carbohydrates (less than 50 g/d).

### 2.2. Selection Criteria

Articles were included in the meta-analysis according to the Participants, Intervention, Comparison, Outcomes, and Study design (PICOS) principle (Table 1). The inclusion criteria were as follows: (a) studies written and published in English; (b) the participants were patients diagnosed with T2DM in terms of glycated hemoglobin (HbA1c) or fasting glucose; (c) the participants were overweight with a body mass index (BMI) of not less than 25 kg/m^2^; (d) the dietary intervention was a ketogenic diet (the intervention group) alongside other types of diets (the control group); (e) the main outcomes included body weight change, glycemic control, and lipid profile; and (f) studies were RCTs. Alternatively, studies were excluded if: (a) they were case reports, meta-analyses, or reviews; (b) they were animal studies; (c) they had no control group; and (d) they had no or insufficient data for calculating mean differences and standard errors, or 95% confidence intervals before and after interventions.

### 2.3. Data Extraction and Quality Assessment

Two reviewers independently screened the titles and abstracts, based on the aforementioned inclusion and exclusion criteria. Full texts were then retrieved and carefully reviewed. Any discrepancies and controversies between them were evaluated by a third author, and resolved by consensus. Data extraction was also independently performed by two reviewers using Microsoft Excel, with missing raw data obtained from the corresponding authors via email. Information retrieved included the name of the first author, year of publication, country, study design, type and duration of intervention, inclusion and exclusion criteria, number of patients, and outcomes. The outcomes are mainly body composition parameters (body weight, BMI, and waist circumference), glycemic control (fasting glucose, fasting insulin, HbA1c, and homeostatic model assessment index of insulin resistance (HOMA-IR)), and lipid profiles (total cholesterol, high-density lipoprotein (HDL), low-density lipoprotein (LDL), and triglycerides).

The quality of the included RCTs was assessed according to the recommendations of the Cochrane Handbook. These were based on the following processes: random sequence generation, allocation concealment, blinding participants and personnel, blinding of outcome assessment, incomplete outcome data, selective reporting, and other bias. Consequently, the risk of bias in randomized studies is classified into three scores, namely: “low”, “high”, and “unclear”. Any disagreements during the analysis of the risk of bias and quality of evidence were resolved by consensus, involving a third reviewer.

### 2.4. Statistical Analysis

Statistical analyses were performed using Review Manager software (Version 5.4, the Cochrane Collaboration). Briefly, sample sizes, means and standard deviations (SD) of continuous variables, before and after intervention, were extracted from each group and presented as the means ± SD (M ± SD). The effects of ketogenic diets on overweight T2DM patients were estimated by weighted standardized mean differences (SMDs), with corresponding 95% confidence intervals (CIs) for each selected study. Heterogeneity among studies was quantitatively evaluated by Cochrane’s Q test and *I*^2^ test. The *I*^2^ value, with a value >50%, is considered high heterogeneity based on a random-effect model. Otherwise, a fixed-effect model was implemented. All tests were two-tailed, and a statistically significant difference was considered at *p* < 0.05. Begg’s funnel plots and Egger’s linear regression tests were used for the detection of publication bias, and a symmetric funnel plot is considered a low risk of publication bias.

## 3. Results

### 3.1. Study Selection

**Figure 1:**
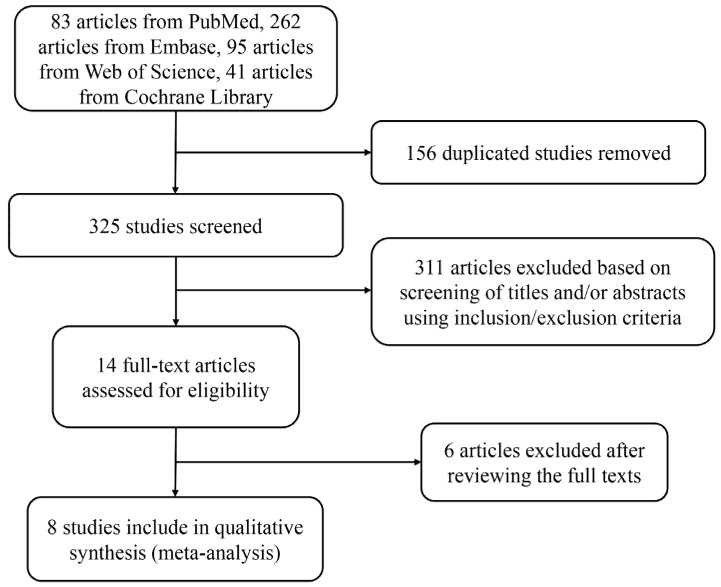
Flow chart of literature selection.

A summary of study selection in this meta-analysis is presented using the flow chart shown in Figure 1. Initial screening of the aforementioned databases resulted in 481 articles, with 83, 262, 95, and 41 articles from PubMed, Embase, Web of Science, and Cochrane Library, respectively. Next, 156 duplicated studies were excluded, and the remaining 325 articles, whose titles and abstracts were subsequently screened, were included according to the aforementioned selection criteria. Among them, 14 full-text articles remained for eligibility assessment. Finally, eight RCTs remained for meta-analysis after carefully reviewing the full texts [18,19,20,21,22,23,24,25].

### 3.2. Study Characteristics

Details of the eight eligible studies are shown in Table 2. Briefly, these RCTs included a total of 611 participants diagnosed with T2DM, of which 331 participants were administered ketogenic diets comprising a daily dietary intake of carbohydrates less than 50 g. The intervention duration varied from 3 months to 2 years. Notably, four trials were conducted in the USA, two in Australia, and one each in Spain and Kuwait. All eight studies reported the effects of post-intervention of ketogenic diets versus baseline on body weight change, glycemic control, and lipid profiles in overweight T2DM patients.

### 3.3. Quality of the Included Trials

**Figure 2:**
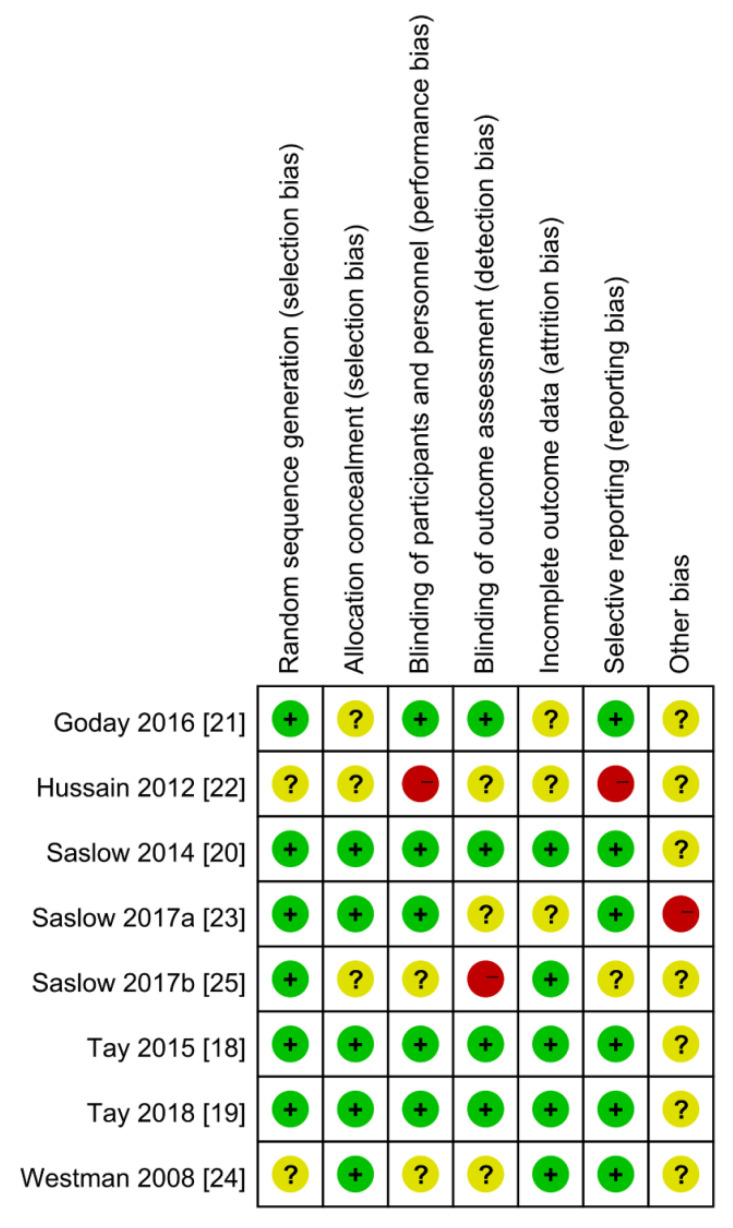
Risk of bias summary.

A summary of the risk of bias for each RCT is shown in Figure 2. One of the RCTs exhibited a high risk of performance bias, while another had a high risk of reporting bias because data on glycemic control and lipid profile are presented as linear graphs. Moreover, one study had a high risk of detection bias, while another had a high risk of other bias due to the involvement of a Scientific Advisory Board. Nevertheless, the included studies were of high quality, with an acceptable risk of bias.

### 3.4. Effects of Ketogenic Diet on Body Weight Change

**Figure 3:**
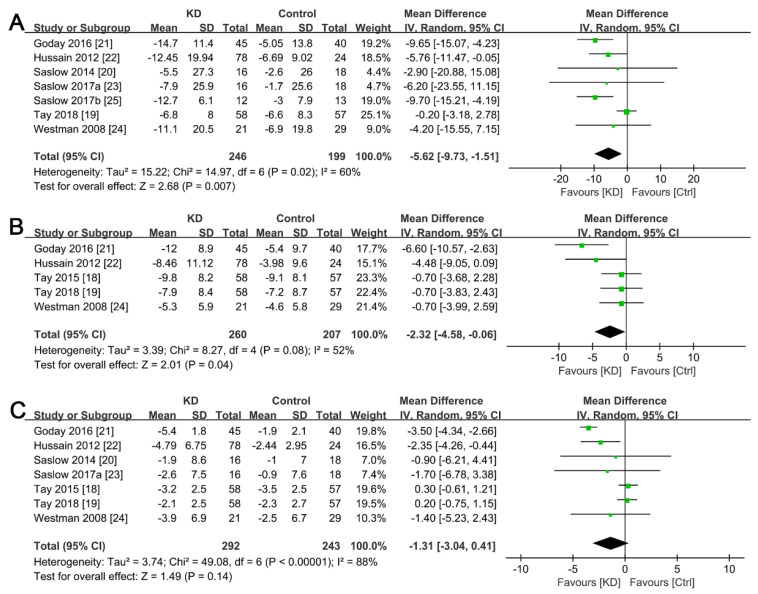
Forest plots for the effects of post-intervention versus baseline on weight change in overweight T2DM patients. (**A**) Changes in body weight; (**B**) Changes in waist circumference; (**C**) Changes in BMI. The green squares represent individual effect sizes, and the black diamonds represent pooling effect sizes.

All eight studies reported parameters in body weight change, with the results demonstrating that T2DM patients exposed to a ketogenic diet were more likely to record a higher body weight loss (SMD, −5.63; 95% CI, −9.76 to −1.49; *I^2^* = 60%; moderate heterogeneity, Figure 3A) and a reduction in waist circumference (SMD, −2.32; 95% CI, −4.58 to −0.06; *I^2^* = 52%; moderate heterogeneity, Figure 3B) when compared to those on other types of diets. Notably, we found no statistical significance in SMD of BMI reduction (*p* = 0.14, Figure 3C).

### 3.5. Effects of Ketogenic Diet on Glycemic Control

**Figure 4:**
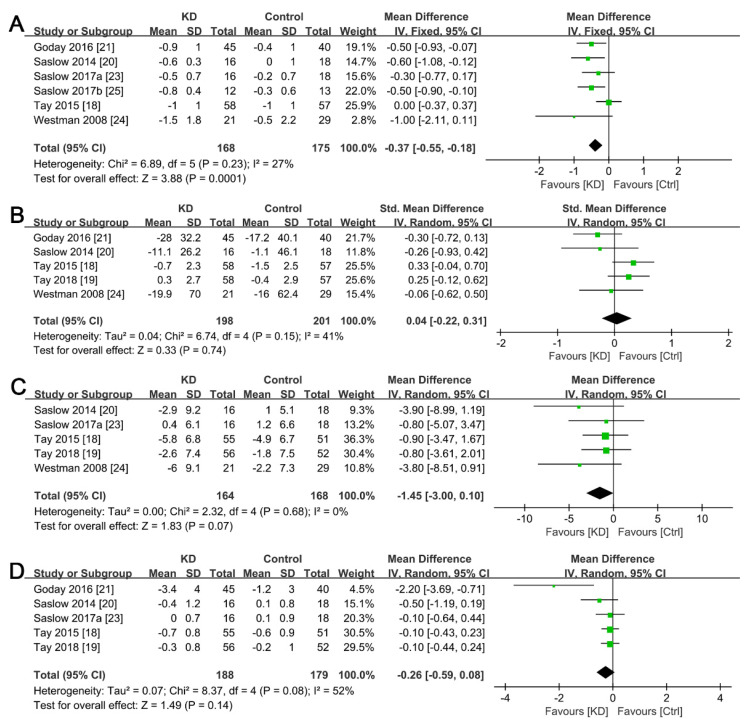
Forest plots for the effects of post-intervention versus baseline on glycemic control in overweight T2DM patients. (**A**) Changes in HbA1c; (**B**) Changes in fasting glucose; (**C**) Changes in fasting insulin; (**D**) Changes in HOMA-IR. The green squares represent individual effect sizes, and the black diamonds represent pooling effect sizes.

Seven of the eight trials included studies reporting glycemic parameters, including fasting glucose, HbA1c, fasting insulin, and HOMA-IR. With regard to HbA1c, we observed a slightly higher decrease between the ketogenic and non-ketogenic diet groups (SMD, −0.38; 95% CI, −0.61 to −0.16; *I*^2^ = 27%; low heterogeneity, Figure 4A) at baseline and post-intervention. However, we found no significant differences in the overall effect for fasting glucose (*p* = 0.74, Figure 4B), fasting insulin (*p* = 0.07, Figure 4C), and HOMA-IR (*p* = 0.14, Figure 4D) between the intervention and control groups. Notably, the reduction in fasting insulin approached borderline significance in favor of the ketogenic diet group, albeit with no significant heterogeneity (*I*^2^ = 0, Figure 4C).

### 3.6. Effects of Ketogenic Diet on Lipid Profiles

**Figure 5:**
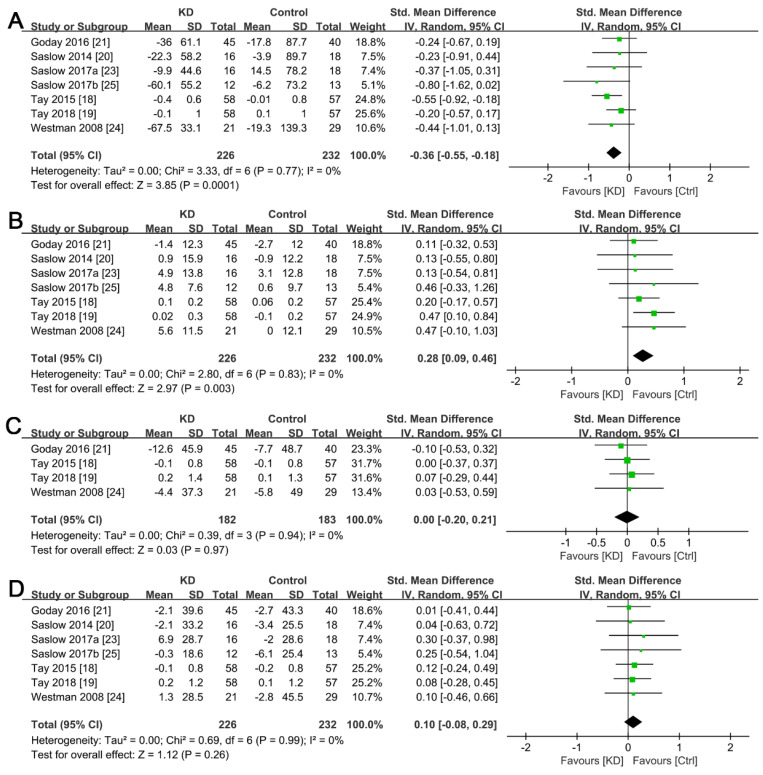
Forest plots for the effects of post-intervention versus baseline on lipid profiles in overweight T2DM patients. (**A**) Changes in fasting triglycerides; (**B**) Changes in HDL; (**C**) Changes in total cholesterol; (**D**) Changes in LDL. The green squares represent individual effect sizes, and the black diamonds represent pooling effect sizes.

The results from subgroup analysis, in the seven studies that reported lipid profiles, revealed that the ketogenic diet was associated with a significantly higher reduction in triglyceride levels (SMD, −0.36; 95% CI, −0.55 to −0.18; *I^2^* = 0%; homogeneity, Figure 5A) and an increase in HDL levels (SMD, 0.28; 95% CI, 0.09 to 0.46; *I*^2^ = 0%; homogeneity, Figure 5B). However, we found no significant differences with regard to changes in total cholesterol and LDL levels between the ketogenic and non-ketogenic diet groups (*p* = 0.97 and *p* = 0.26, respectively, Figure 5C,D), despite a lack of heterogeneity in both lipid parameters between the groups.

## 4. Discussion

In the present meta-analysis, the results from eight studies reporting the effect of a ketogenic diet on patients with T2DM revealed that this diet is an effective intervention for lowering body weight and glycemic levels, as well as improving lipid profiles in overweight diabetic patients. Notably, the ketogenic diet exhibited excellent benefits in reducing body weight, waist circumference, HbA1c, and triglycerides, as well as increasing HDL.

Obesity, which is highly prevalent in patients with T2DM, has been associated with chronic inflammation statuses, such as mitochondrial dysfunction, endoplasmic reticulum stress, and hyperinsulinemia [26]. Body weight control is considered an effective intervention strategy for attenuating insulin resistance induced by obesity [27,28]. Numerous meta-analyses and reviews have investigated the effect of a ketogenic diet on body weight control and found that nutritional ketosis is a beneficial process in body weight management [29,30,31]. Similarly, the results from the present study demonstrated that a ketogenic diet is a significantly superior intervention over other diets with regard to reducing body weight in obese T2DM patients (Figure 3). Additionally, ketogenic diet-based intervention was associated with a significant reduction in waist circumference, a parameter of central obesity that has been shown to be an important risk factor for the progression and prognosis of diabetes and related complications [32]. Moreover, there was no significant difference between the groups in terms of BMI reduction, which is the most commonly used parameter for assessing obesity. The observed significant reduction in body weight after ketogenic diet intervention (Figure 3A) might be due to attenuation of decreased resting energy expenditure modulation, as previously reported [33]. However, the exact mechanisms of a ketogenic diet on body weight loss remain unclear. For instance, some mechanistic studies have indicated that a ketogenic diet can suppress appetite by either directly affecting ketone bodies [34] or regulating appetite control hormones [35]. On the other hand, other evidence has demonstrated a great metabolic efficiency of fat consumption by reducing the resting respiratory quotient in patients assigned to a ketogenic diet [36]. Moreover, other findings have suggested that the high consumption of fat after ketogenic diets might be due to reduced lipogenesis, increased lipolysis, and increased metabolic costs of gluconeogenesis [37]. Regardless of the underlying mechanism of action, it is evident that a ketogenic diet exerts a remarkable effect on body weight loss in overweight patients with T2DM.

Extreme restriction of daily dietary intake of carbohydrates causes a decline in the absorption of monosaccharides, reduces blood glucose levels, and limits blood glucose fluctuations, indicative of positive regulation of glucose metabolism [38,39]. Consequently, this phenomenon may contribute to the benefit of a ketogenic diet on glycemic control in T2DM patients. HbA1c levels can reflect average blood glucose concentrations in the past 2–3 months in patients with T2DM; hence, it has been recommended as an effective parameter for monitoring long-term glycemic regulation and a risk predictor [40]. The results of the present meta-analysis revealed that the consumption of a ketogenic diet was likely to induce a greater reduction in HbA1c in overweight patients with T2DM than in those under other types of diets (Figure 4). Moreover, the intervention duration of the included studies was at least 3 months; thus, the change in HbAlc between baseline and post-intervention can be used to effectively evaluate the efficacy of a ketogenic diet for controlling blood glucose levels. This result was consistent with those reported in other systemic reviews and meta-analyses, in which a ketogenic diet was found to remarkably improve glycemic profiles [41]. Furthermore, accumulating evidence has demonstrated a strong relationship between insulin resistance and the ketogenic diet [42,43]. A mild increase in ketosis in peripheral blood, induced by a ketogenic diet, might improve peripheral insulin sensitivity, relieve hyperinsulinemia-related stress, reduce external insulin requirements, and inhibit its secretion, thereby improving glycemic profiles and mitigating insulin resistance [44]. Moreover, ketone bodies can increase the concentration of intracellular glucose and generate metabolic effects similar to those of insulin, but without activating the insulin signaling pathway, which allows for a therapeutic effect of mild ketosis in insulin resistance states [42]. In the present meta-analysis, although we found no evidence that such an intervention could significantly affect fasting insulin levels in diabetic patients, the reduction in insulin levels approached borderline significance in favor of the ketogenic diet group, with excellent homogeneity, suggesting that this diet has potential health benefits on insulin profiles.

The majority of daily calories are from fat intake in a ketogenic diet, and increasing fat consumption may improve lipid profiles in obese diabetic patients, as previously described [45]. Interestingly, the consumption of a ketogenic diet could result in the improved lipid profiles and remarkably improved glucose metabolism [41]. In the current meta-analysis, seven out of the eight clinical trials reported the analysis of lipid profiles, including triglycerides, total cholesterol, HDL, and LDL. Our results revealed statistically significant changes in triglycerides and HDL levels after ketogenic diet consumption, which may be attributed to the inclusion criteria used to select the participants (Figure 5). Notably, the recruited individuals were overweight T2DM patients, with a BMI > 25 kg/m^2^, and who’s glycemic and lipid profiles were in a total mess. In addition, the basal lipid biomarkers were much higher than that of healthy controls, suggesting the possibility that a strict diet intervention might induce significant changes [46]. Since the components of diabetic dyslipidemia were mostly related to insulin resistance [47], the improvement of dysfunctional lipid profiles could reduce the risks of cardiovascular diseases in diabetic patients [48]. Although we did not explore the cardioprotective effects of a ketogenic diet in overweight patients with T2DM, the potential therapeutic effects of such dietary management on cardiovascular diseases cannot be ignored. Previous mechanistic studies have shown that the improvement of dyslipidemia induced by a ketogenic diet may not only benefit the regulation of insulin sensitivity, but also control and prevent the occurrence and progression of related complications [49,50].

Similarly, this study has some limitations. First, only eight studies were enrolled in our meta-analysis, owing to a limited number of studies that have evaluated the effect of a ketogenic diet on T2DM patients. Second, the included studies did not have some data on body weight change, glycemic control, or lipid profile, which may generate biases toward the overall effect. Third, none of these studies were carried out in East Asian countries, and the included individuals were more likely to be Caucasians, which may generate population bias. Fourth, although the Cochrane risk approach is the most recommended method for analyzing the risk of bias in RCTs, it has been associated with limitations, especially when assessing some complicated and complex interventions involved in behavior or lifestyle [51].

## 5. Conclusions

The results of the current meta-analysis reveal that ketogenic diet intervention has remarkable benefits on body weight and glycemic control, as well as the improvement of lipid profiles in overweight T2DM patients. Specifically, a ketogenic diet can reduce body weight, waist circumference, HbA1c, and triglycerides, and increase HDL levels. Thus, the ketogenic diet intervention for overweight T2DM patients could be considered. Moreover, the ketogenic diet could reveal more benefits to the improved body compositions for mitigating the development and progression of T2DM due to overweight or obesity by lowering body weight, reducing glycemic levels, and improving lipid profiles. In the future, comprehensive mechanistic studies need to be conducted to underpin associations between ketogenic diets and overweight patients with T2DM, and even confirmed by experimental exploration.

## Figures and Tables

**Table 1:** PICOS criteria for inclusion and exclusion of studies.

Parameters	Inclusion Criteria
Population	Population>Overweight T2DM patients
Intervention	Ketogenic diet; very low-carbohydrate diet
Comparison	Any comparisons
Outcomes	Body weight change, glycemic control, lipid profile
Study design	Randomized controlled trials

**Table 2:** Characteristics of included studies.

First Author/Year	Country	Study Design	Intervention Duration	Inclusion Criteria	Exclusion Criteria	Interventions	N	Outcomes
Goday (2016) [21]	Spain	RCT	4 months	Age: 30–65 years old; BMI: 30–35 kg/m^2^; T2DM	T2DM duration longer than 10 years; insulin therapy; HbA1c ≥ 9%; fasting C-peptide < 1 ng/mL. In addition: impaired renal or liver function, alcohol intake ≥ 40 g/d for men and ≥ 24 g/d for women, pregnancy, lactation, sever eating or psychiatric disorder.	VLCKD: <50 g/d carbohydrates	45	body weight, BMI, waist circumference, fasting plasma glucose, HbA1c, fasting insulin, HOMA-IR, total cholesterol, TG, LDL, HDL
						LCD: a daily energy restriction of 500–1000 kcal, <30% fat, 10–20% protein, 45–60% carbohydrates.	44	
Saslow (2014) [20]	USA	RCT	3 months	Age: >18 years old; BMI: ≥25 kg/m^2^; T2DM (HbAc1 ≥ 6.5) or prediabetes (HbAc1 ≥ 6.0)	insulin or more than 3 glucose-lowering agents; oral glucocorticoids or weight loss medications; pregnancy; breastfeeding; weight loss surgery; vegan	LCKD: 20–50 g/d carbohydrates	16	HbA1c, LDL, HDL, TG, fasting glucose and insulin, HOMA-IR, body weight, BMI, waist circumference
						MCCRD: 45–50% carbohydrates.	18	
Saslow (2017a) [23]	USA	RCT	12 months	Age: >18 years old; BMI: ≥ 25 kg/m^2^; T2DM (HbAc1 ≥ 6.5) or prediabetes (HbAc1 ≥ 6.0)	insulin or more than 3 glucose-lowering agents	LCKD: 20–50 g/d carbohydrates	16	HbA1c, LDL, HDL, TG, fasting glucose and insulin, HOMA-IR, body weight, BMI, waist circumference
						MCCRD: 45–50% carbohydrates.	18	
Saslow (2017b) [25]	USA	RCT	32 weeks	Age ≥ 18 years old; BMI ≥ 25 kg/m^2^; T2DM (HbA1c 6.5–9.0)	any diabetes medication other than metformin	LCKD: 20–50 g/d carbohydrates	12	HbA1c, LDL, HDL, TG; body weight, BMI, waist circumference
						American Diabetes Associations’ “Create Your Plate” diet	13	
Tay (2015) [18]	Australia	RCT	52 weeks	Age: 35–68 years old; BMI: 26–45 kg/m^2^; T2DM (HbA1c ≥ 7.0 and/or antidiabetic treatment)	T1DM; impaired renal or liver function; overt endocrinopathy; history of malignancy; respiratory disease, gastrointestinal disease, or CVD; pregnancy or lactation; history of or current eating disorder or smoking.	LCD: 14% carbohydrates (<50 g/d), 28% protein, 58% fat (35% monounsaturated fat and 13% polyunsaturated fat)	58	HbA1c, LDL, HDL, total cholesterol, TG, fasting glucose and insulin, HOMA-IR, body weight, BMI, waist circumference
						HCD: 53% carbohydrates, 17% protein, 30% fat (15% monounsaturated fat and 9% polyunsaturated fat)	57	
Tay (2018) [19]	Australia	RCT	2 years	Age: 35–68 years old; BMI: 26–45 kg/m^2^; T2DM (HbA1c ≥ 7.0 and/or antidiabetic treatment)	T1DM; renal, hepatic, respiratory, gastrointestinal, or cardiovascular disease; history of malignancy; any significant endocrinopathy; pregnancy/lactation; history of or current eating disorder or smoking.	LCD: 14% carbohydrates (<50 g/d), 28% protein, 58% fat	58	HbA1c, LDL, HDL, total cholesterol, TG, fasting glucose and insulin, HOMA-IR, body weight, BMI, waist circumference
						HCD: 53% carbohydrates, 17% protein, 30% fat	57	
Westman (2008) [24]	USA	RCT	24 weeks	Age: 18–65 years old; BMI: 27–50 kg/m^2^; T2DM > 1 year (HbA1c > 6.0);	unstable or serious medical condition; significant co-morbid illnesses such as liver disease, kidney disease, cancer; pregnancy; or nursing mothers.	LCKD: <20 g/d carbohydrates	48	HbA1c, fasting glucose, fasting insulin, body weight, BMI, waist circumference
Hussain (2012) [22]	Kuwait	RCT	24 weeks	Age ≥ 18 years; BMI > 25 kg/m^2^; fasting serum glucose > 6.9 mM.	renal insufficiency, liver disease, or unstable cardiovascular disease.	LCKD: <20 g/d carbohydrates	78	body weight, BMI, waist circumference, HbA1c, fasting glucose, TG, total cholesterol, LDL, HDL.

**Note:** BMI: body mass index; T2DM: type 2 diabetes mellitus; TG: triglycerides; HbA1c: glycosylated hemoglobin; HOMA-IR: homeostasis model assessment of insulin resistance; VLCKD: very low-carbohydrate ketogenic diet; LCKD: low-carbohydrate ketogenic diet; MCCRD: medium carbohydrate, low fat, calorie-restricted diet; HCD: high-carbohydrate diet; LCD: low-carbohydrate diet; LGID: low-glycemic index diet.

## Data Availability

The data that support the findings of this study are available from the corresponding author upon reasonable request.
